# Biocompatibility and Immune Response of a Newly Developed Volume-Stable Magnesium-Based Barrier Membrane in Combination with a PVD Coating for Guided Bone Regeneration (GBR)

**DOI:** 10.3390/biomedicines8120636

**Published:** 2020-12-20

**Authors:** Larissa Steigmann, Ole Jung, Wolfgang Kieferle, Sanja Stojanovic, Annica Proehl, Oliver Görke, Steffen Emmert, Stevo Najman, Mike Barbeck, Daniel Rothamel

**Affiliations:** 1 Department of Periodontics and Oral Medicine, School of Dentistry, University of Michigan, Ann Arbor, MI 48109, USA; lasteigm@umich.edu; 2 Clinic and Policlinic for Dermatology and Venereology, University Medical Center Rostock, D-18057 Rostock, Germany; ole.tiberius.jung@googlemail.com (O.J.); steffen.emmert@med.uni-rostock.de (S.E.); 3 TS TriboSystems GmbH, D-88250 Weingarten, Germany; w.kieferle@tribosystems.de; 4 Department of Biology and Human Genetics, Faculty of Medicine, University of Niš, 18000 Niš, Serbia; s.sanja88@gmail.com (S.S.); stevo.najman@gmail.com (S.N.); 5 Department for Cell and Tissue Engineering, Scientific Research Center for Biomedicine, Faculty of Medicine, University of Niš, 18000 Niš, Serbia; 6 Research Department, BerlinAnalytix GmbH, D-12109 Berlin, Germany; annica.proehl@berlinanalytix.com; 7 Department of Ceramic Materials, Chair of Advanced Ceramic Materials, Institute for Materials Science and Technologies, Technical University Berlin, D-10623 Berlin, Germany; oliver.goerke@ceramics.tu-berlin.de; 8 Department of Oral and Maxillofacial Plastic Surgery, Evangelic Johanniter Hospital Bethesda Mönchengladbach, D-41061 Mönchengladbach, Germany; Daniel.Rothamel@mg.johanniter-kliniken.de; 9 Department of Oral and Maxillofacial Plastic Surgery, Heinrich-Heine Universität Düsseldorf, D-40225 Düsseldorf, Germany

**Keywords:** magnesium implant, cytocompatibility, biocompatibility, barrier membrane, dentistry, guided bone regeneration, macrophage

## Abstract

To date, there are no bioresorbable alternatives to non-resorbable and volume-stable membranes in the field of dentistry for guided bone or tissue regeneration (GBR/GTR). Even magnesium (Mg) has been shown to constitute a favorable biomaterial for the development of stabilizing structures. However, it has been described that it is necessary to prevent premature degradation to ensure both the functionality and the biocompatibility of such Mg implants. Different coating strategies have already been developed, but most of them did not provide the desired functionality. The present study analyses a new approach based on ion implantation (II) with PVD coating for the passivation of a newly developed Mg membrane for GBR/GTR procedures. To demonstrate comprehensive biocompatibility and successful passivation of the Mg membranes, untreated Mg (MG) and coated Mg (MG-Co) were investigated in vitro and in vivo. Thereby a collagen membrane with an already shown biocompatibility was used as control material. All investigations were performed according to EN ISO 10993 regulations. The in vitro results showed that both the untreated and PVD-coated membranes were not cytocompatible. However, both membrane types fulfilled the requirements for in vivo biocompatibility. Interestingly, the PVD coating did not have an influence on the gas cavity formation compared to the uncoated membrane, but it induced lower numbers of anti-inflammatory macrophages in comparison to the pure Mg membrane and the collagen membrane. In contrast, the pure Mg membrane provoked an immune response that was fully comparable to the collagen membrane. Altogether, this study shows that pure magnesium membranes represent a promising alternative compared to the nonresorbable volume-stable materials for GBR/GTR therapy.

## 1. Introduction

The regenerative principle of guided bone and tissue regeneration (GBR/GTR) is based on cell exclusivity through barrier membranes [1]. Until now, only nonresorbable membranes mostly based on compounds like dense polytetrafluoroethylene (dPTFE) or expanded polytetrafluoroethylene (e-PTFE) provide volume stability in combination with titanium meshes as so-called titanium-reinforced PTFE membranes [2,3]. However, such materials require a second surgical intervention for extraction and may lead to wound opening and bacterial infiltration with subsequent infection [4]. Up to date, this feature is exclusively reserved for this membrane type making its application indispensable even for multidimensional bony defects.

To overcome the limitations of the currently used non-resorbable barrier membranes, a new generation of membranes has been introduced, combining the advantages of resorbability with volume stability. An initial step in this direction was reported in a publication by Barbeck et al. that showed the good biocompatibility of a new bioresorbable barrier membrane composed of a hydrofluoric acid (HF)-treated magnesium (Mg) mesh embedded in a native collagen membrane for volume stable indications [5,6]. In this study, the HF-treatment was applied for passivation of the implanted magnesium meshes to slow down its biodegradation as it is known that the application of pure magnesium implants may lead to their rapid uncontrolled degradation along with hydrogen gas evolution and related tissue incompatibilities [5]. In this context, a variety of coatings have been analyzed for their suitability to enable the safe application of magnesium implants [5,7,8]. Interestingly, most of the coatings are based on (calcium) phosphate compounds and are thus not suitable for combination with a magnesium-based barrier membrane due to their brittleness and inflexibility [7,9,10]. An appropriate alternative method is coatings made via physical vapor deposition (PVD), which produces thin films on the surface of biomaterials [7,11,12]. This coating technique is mainly used to improve the optical, mechanical or tribological characteristics of different materials such as surgical instruments [7,11,12]. The technique ion implantation (II) includes the bombardment of ionized species and their implantation into the first atomic layers of a solid and can be combined with different other coating techniques [13,14]. Thus, both techniques allow for surface modifications using different implanted ions and an effect on material properties such as mechanical characteristics, antibacterial properties, allergenicity or also corrosion resistance. In this context, it has been used to produce different coatings on the surfaces of magnesium-based implants in order to improve their corrosion resistance [15].

Additionally, it has been suggested that the next generation of biomaterials used for (bone) tissue regeneration should also provide immunoregulative functionalities [16]. Thus, a biomaterial should provide a special composition of physicochemical material characteristics that allow to modify specific immune responses and support tissue regeneration on a molecular level. In this context, it has been revealed that especially macrophages, but also cell types such as resident stem cells are key players in the inflammatory tissue responses to a biomaterial and the regeneration [17,18,19]. These cell types should be influenced by the biomaterial to act as anti-inflammatory and immunomodulatory elements in the local environment with the aim to trigger bone replacement and optimally processes osteoinduction or osteogenesis [16,20].

The present study was conducted to test the functionality of the PVD coating method to improve the biocompatibility of a newly developed volume-stable Mg barrier membrane. After cytocompatibility assessment, the subcutaneous implantation model in BALB/c mice was used to analyze the tissue reactions to this new membrane in comparison to an uncoated magnesium membrane and an already available and manifoldly examined collagen-based barrier membrane as already published [2,6,21,22]. Moreover, the immune responses were comparatively analyzed based on the detection of M1- and M2-macrophages based on a previously described protocol [2,23,24]. Established histological (immuno-) histochemical staining methods as well as histopathological and histomorphometric procedures were used for the execution of the present in vivo study [24,25,26,27,28].

## 2. Experimental Section

### 2.1. Ion implantation and Physical Vapor Deposition (PVD)

The Mg membranes were passivated by means of ion implantation under argon atmosphere followed by PVD treatment using a specially constructed coating system from the company Ts TriboSystems (Weingarten, Germany).

The deposition process steps were as follows: (a) vacuum level before starting the evaporation of Cr: 1 × 10^−5^ mbar, (b) heating up to 400–450 °C, (c) argon etching for 30 min, (d) deposition of a thin chromium interfacial layer and (e) deposition of CrN layer. After the deposition process, the treated and untreated Mg-membranes were cleaned in an ultrasonic bath and then dried and packaged under sterile conditions within a laminar flow chamber.

### 2.2. Cytocompatibility Analysis

Cytocompatibility analysis was conducted in accordance with the ISO 10993-5/-12 as described in our previous publications [5,29,30,31]. Therefore, the process is briefly summarized:

#### 2.2.1. Reference Materials (Positive and Negative Controls)

RM-A (Hatano Research Institute, Food and Drug Safety Center, Japan) was used as positive control. This reference material induces a given level of cytotoxicity, and it is composed of a polyurethane film containing 0.1% zinc diethyldithiocarbamate [29]. Cell wells and titanium grades 4 and 5 were used as negative controls. All reference materials were prepared and treated in the same way as the experimental samples.

#### 2.2.2. Cell Culture and Conditions

L-929 mouse fibroblasts were obtained from the European Collection of Cell Culture, ECACC (Salisbury, UK). Cells were cultured in cell culture medium under standard cell-cultured conditions. Cells were passaged at around 80% confluency. As cell culture medium, minimum essential medium (MEM) supplemented with 10% fetal bovine serum, penicillin/streptomycin (100 U/mL each) (all from Life Technologies, Carlsbad, CA, USA) and 4 mM L-glutamine (Sigma-Aldrich, St. Louis, MO, USA) was used at 37 °C, 5% CO_2_ and 95% humidity (further referred as cell culture conditions).

#### 2.2.3. Extract Analysis

##### Extraction of the Biomaterials and the Reference Materials

For the cytotoxicity tests, the extract dilution method was used according to the respective DIN ISO 10993-5/-12 norms. Test and control samples were extracted for 72 h at a surface to volume ratio of 3 cm^2^/mL in cell culture medium under cell culture conditions. After extraction, extracts were centrifuged and further utilized in the viability and toxicity assays.

##### Assay Procedure

A total of 100 μL of the extract was given to 96-well plates seeded with 1 × 10^4^ L929 cells/well in 100 μL cell culture medium. The wells were incubated under cell culture conditions for 24 h. After 24 h, sodium 3,3′-[1(phenylamino)carbonyl]-3,4-tetrazolium]-3is(4-methoxy-6-nitro) benzene sulfonic acid hydrate (XTT, Roche Diagnostics, Mannheim, Germany) assay, bromodeoxyuridine (BrdU, Roche Diagnostics, Mannheim, Germany) assay and lactate dehydrogenase (LDH, BioVision, Milpitas, CA, USA) assay were conducted according to the manufacturer’s instructions.

#### 2.2.4. Live-Dead Staining

For direct cell viability, all materials were seeded with 2.4 × 10^5^ cells in 1 mL cell culture medium in 12-well plates (surface-area/medium ratio: 5.65 cm^2^/mL) for 24 h under cell culture conditions. Staining was achieved by adding 50 µL of the propidium iodide (PI) stock solution (2 mg PI in 1 mL PBS) and 8 µL of the fluorescein diacetate (FDA) stock solution (5 mg FDA in 1 mL acetone) to each well. After 3 min incubation time at room temperature, the test samples were rinsed with prewarmed PBS and examined with an upright fluorescence microscope (Nikon ECLIPSE Ti-S/L100, Nikon GmbH, Düsseldorf, Germany).

### 2.3. In Vivo Study

The in vivo study was conducted at the Faculty of Medicine (University of Niš, Niš, Serbia) following a previously described protocol following authorization by the local ethical committee (Faculty of Medicine, University of Niš, Niš, Serbia) on the basis of which the Veterinary Directorate of the Ministry of Agriculture, Forestry and Water Management of the Republic of Serbia issued the decision number 323-07-00278/2017-05/6 (Date: 13/07/2017) [2,5,6,21,22]. Thereby, the experimental animals were housed using standard conditions, i.e., water ad libitum, artificial light and regular rat pellets, in combination with standard pre- and postoperative care.

Briefly, the in vivo study was conducted using 30 female, 6–8 week-old BALB/c mice that were obtained from the Military Medical Academy (Belgrade, Serbia). These experimental animals were divided into three study groups with 10 animals per group and 5 animals per time point (*n* = 5), i.e., 10 and 30 days. In brief, after anesthesia of the experimental animals by means of intraperitoneal injection with 10 mL ketamine (50 mg/mL) combined with 1.6 mL xylazine (2%), the subscapular region of the animals was initially shaved and disinfected. Subsequently, an incision was made, followed by blunt preparation of a subcutaneous pocket in which the biomaterials were inserted. Finally, suturing using a standard suture material was conducted.

The explantation included the initial euthanasia of the experimental animals by means of an overdose of the previously described anesthetics. Afterward, the tissue within the implantation area, including the (remnants of the) biomaterials and the peri-implant tissue, were removed and directly inserted into fixation solution (4% formalin solution) for 24 h to be ready for further histological preparation.

#### 2.3.1. Histological and Immunohistochemical Staining Methods

Initially, dehydration by means of increasing alcohol concentrations and a final xylol treatment followed by paraffin embedding was conducted. For further histological processing, sections with a thickness of 3–5 μm were cut using a rotary microtome (SLEE, Mainz, Germany). Afterward, the histochemical staining methods hematoxylin and eosin (H&E), Masson Goldner, Movat’s pentachrome and Giemsa were applied on three consecutively taken sections. Immunohistochemical detection of the M1- and M2-macrophage subforms was performed by means of antibodies against the hemoglobin scavenger receptor (CD163) and the transcription factor “nuclear factor kappa-light-chain-enhancer” (NF-kB). Workup and slide preparation were standardized in accordance with previously published methods [2,5,6,21,22]. As a preparatory step for antigen demasking, slides were treated with citrate buffer and Tris EDTA pH 8 buffer (Zytomed Systems, Berlin, Germany) for 20 min in a water bath at 96 °C, followed by equilibration and cooling down using TBS-T buffer. To avoid irregular antibody binding, which leads to unspecific background staining, incubation with blocking solution (Zytomed Systems, Berlin, Germany) was inserted before continuing with the respective first antibody for 30 min. Final chromogenic detection was effected by incubation with the secondary antibody (goat anti-rabbit IgG (H + L) secondary antibody, AP, Invitrogen, Carlsbad, CA, USA) and subsequent chromogen exposure with permanent AP-red chromogen (Zytomed Systems, Berlin, Germany) for 10 min at room temperature. Afterward, counterstaining with diluted hematoxylin (Merck KGaA, Darmstadt, Germany) and bluing was conducted.

#### 2.3.2. Histopathological and Histomorphometric Analyses

The histopathological analysis was conducted based on a previously described protocol to investigate the tissue-biomaterial-interactions by means of a light microscope (Axio.Scope.A1, Zeiss, Oberkochen, Germany) [2,5,6,21,22]. These analyses focused on the evaluation of a variety of parameters within the framework of the early and the late tissue response based on the DIN ISO 10993-6 norm package. Microscopic images were made by means of a connected microscope camera (Axiocam 305 color, Zeiss, Oberkochen, Germany) in combination with a computer system running the ZEN Core 3.0 (Zeiss, Oberkochen, Germany) connected to the microscope.

The histomorphometric analyses were done to compare the occurrence of gas cavities within the implantation beds of the membranes and the induction of M1- and M2-macrophages by the three membranes as previously published [5]. In summary, the slides were initially digitized using a specialized scanning microscope combined with an Axio.Scope.A1 microscope, an Axiocam 305 color digital camera, an automatic scanning table (Maerzhaeuser, Wetzlar, Germany) and a PC system with the ZEN Core 3.0 software (all: Zeiss, Oberkochen, Germany). The measurement of the gas cavities included the marking of the total implant area (TIA, in mm^2^) and the gas cavities (in mm^2^), which were subsequently related to obtaining the percentage of the gas formation within the implant beds of the membranes. Additionally, the amounts of the immunohistochemically detected pro- and anti-inflammatory macrophages were related to the total implant area (cells/mm^2^) after their manual counting.

#### 2.3.3. Statistical Analyses

The statistical analysis included an analysis of variance (ANOVA) and a following LSD post hoc test for a comparison of the data from the different study groups by means of the GraphPad Prism 8.1 software (GraphPad Software Inc., La Jolla, CA, USA). Statistical differences were designated as significant if *p*-values were less than 0.05 (* *p* ≤ 0.05) and highly significant if *p*-values were less than 0.01 (** *p* ≤ 0.01) or less than 0.001 (*** *p* ≤ 0.001). Finally, the data were displayed as mean ± standard deviation.

## 3. Results

### 3.1. In Vitro Results

**Figure 1:**
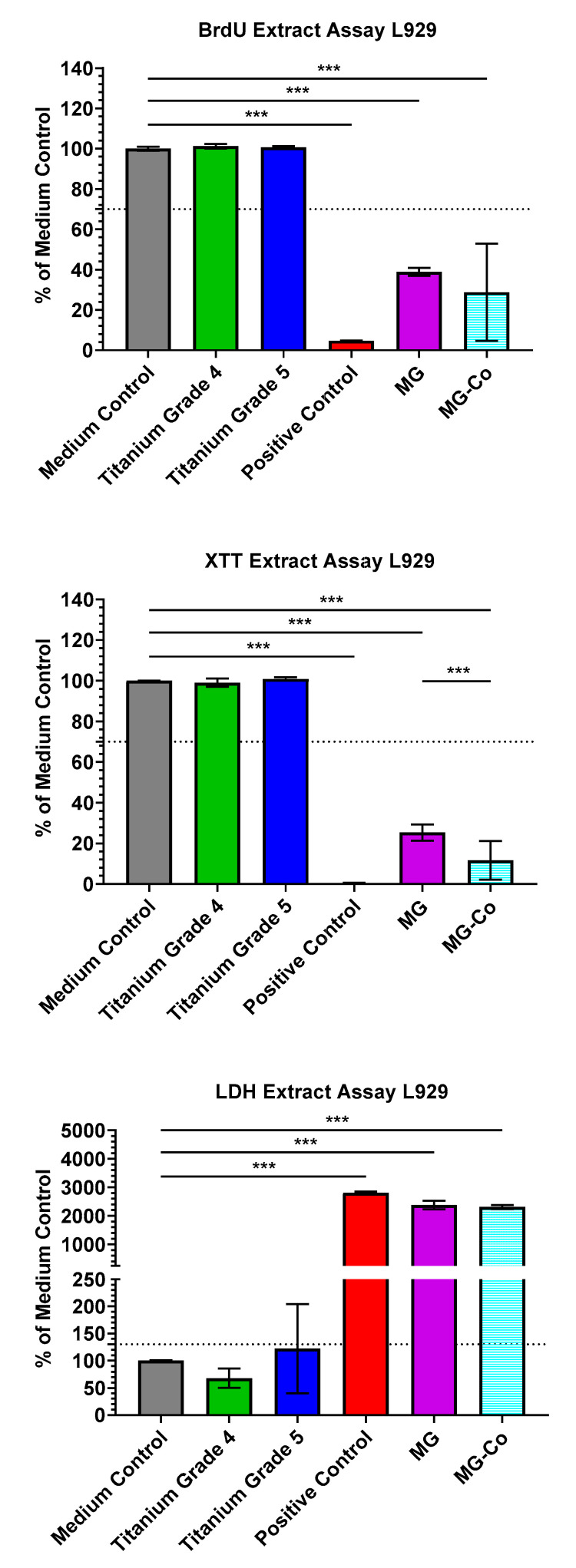
Cytocompatibility of both treated and untreated magnesium variants. Cytotoxicity was measured by LDH-assay; viability was measured by sodium 3,3′-[1(phenylamino)carbonyl]-3,4-tetrazolium]-3is(4-methoxy-6-nitro) benzene sulfonic acid hydrate (XTT) assay, and proliferation was measured by bromodeoxyuridine (BrdU) assay. Means with error bars indicating standard deviations. The dotted line indicates thresholds that should not be exceeded (lactate dehydrogenase (LDH)) or fall below (XTT; BrdU) (*** *p* < 0.001).

In the cytotoxicity tests, values < 130% of the medium control in the cytotoxicity assay (LDH) and values > 70% of the medium control in the proliferation and cell differentiation assays (XTT, BrdU) are within the nontoxic range according to ISO 10993-5:2009 (Figure 1) [5,29,30,31]. Thereby, both treated and untreated magnesium membranes showed values outside these limits in all assays. The values of both membranes more closely approximated the positive control than the other test samples. In both the BrdU and XTT assay, the untreated membrane exhibited higher values than the passivated Mg membrane. In the XTT assay, this difference was highly significant.

**Figure 2:**
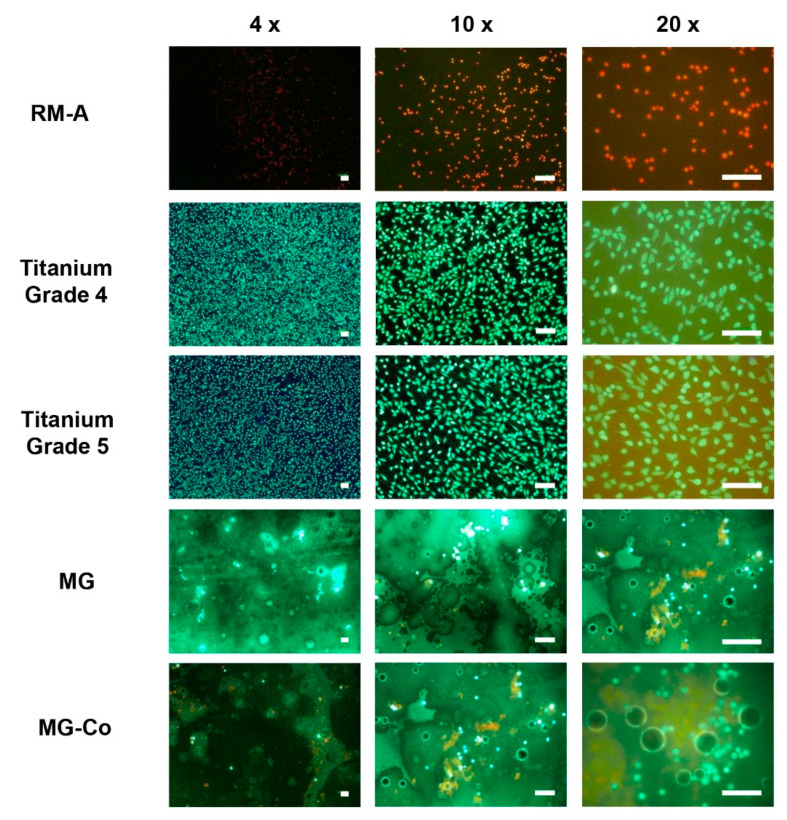
Live–dead staining of the test samples. Pure magnesium membrane = MG, coated magnesium membrane = MG-Co. Green: vital cells; red: dead cells (scale bars = 100 µm).

In concordance with the extract results, live–dead staining revealed similar results (Figure 2). Here, green-colored cells indicate living cells, whereas red-colored cells indicate dead cells. Both titanium controls showed green cells with spindle-shaped cell morphology. In contrast, cells on the positive control were red and rounded. The untreated Mg membrane showed mostly green, non-attached cells and gas bubbles as a sign of active corrosion. In contrast, the treated Mg membrane exhibited mostly dead cells, gas bubbles and dark discoloration.

### 3.2. Histopathological Analyses

**Figure 3:**
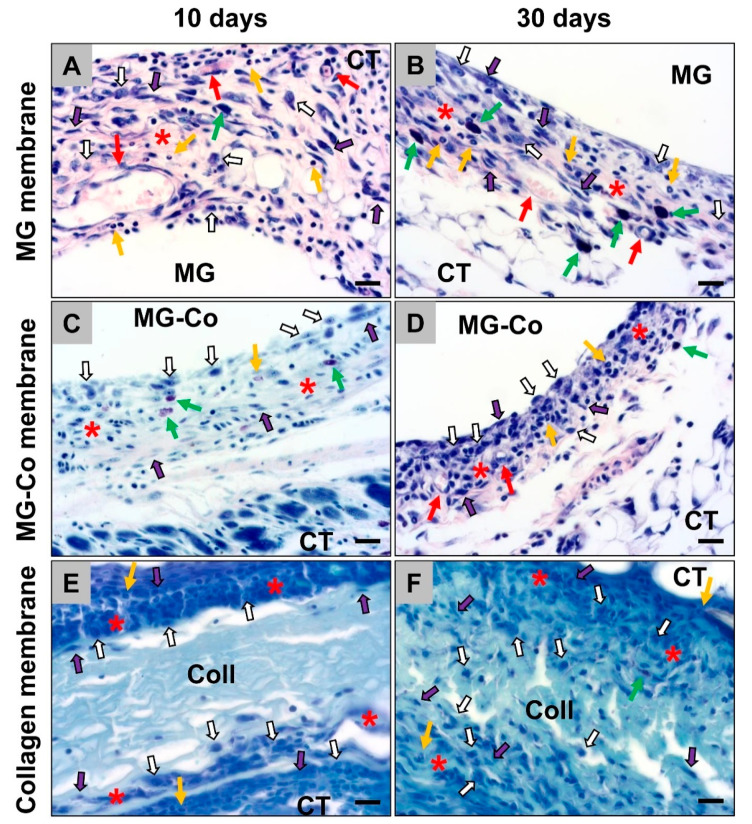
Exemplary histological images of the tissue reactions to the three different membrane types, i.e., the pure magnesium membrane (MG) (**A**,**B**), the coated magnesium membrane (MG-Co) (**C**,**D**) and the collagen membrane (Coll) (**E**,**F**), within the subcutaneous connective tissue (CT) at day 10 and 30 post implantation. Red asterisks = reactive cell multilayer at the material surfaces, white arrows = macrophages, yellow arrows = eosinophils, purple arrows = fibroblasts, green arrows = mast cells, red arrows = blood vessels (Giemsa staining, 400× magnification, scale bars = 10 µm).

The histopathological analysis showed that all membranes induced an inflammatory tissue reaction at day 10 post implantation within their subcutaneous implantation beds (Figure 3). In addition to high numbers of macrophages, higher numbers of (mostly eosinophilic) granulocytes and fibroblasts were also detectable within the cell multilayers at the material–tissue interfaces (Figure 3). Especially in the study group of the coated Mg-membranes, an accumulation of macrophages was observed directly attached to the material surfaces at this early study time point (Figure 3). Moreover, single mast cells were observable within the reactive cell walls, especially in the groups of both magnesium-based barrier membranes (Figure 3). Furthermore, moderate numbers of blood vessels were especially findable within the surrounding tissue of the pure MG membranes (Figure 3).

On day 30 post implantation, still, the aforementioned cell types were found within the implantation beds of all three membrane types (Figure 3). Macrophages were still the dominating cell type within the surrounding tissue at this time point, while also lower numbers of fibroblasts as well as single (eosinophilic) granulocytes and mast cells were detectable (Figure 3). Moreover, moderate numbers of vessels were especially found in the study groups of the magnesium-based membranes in the neighborhood of the material surfaces (Figure 3)

**Figure 4:**
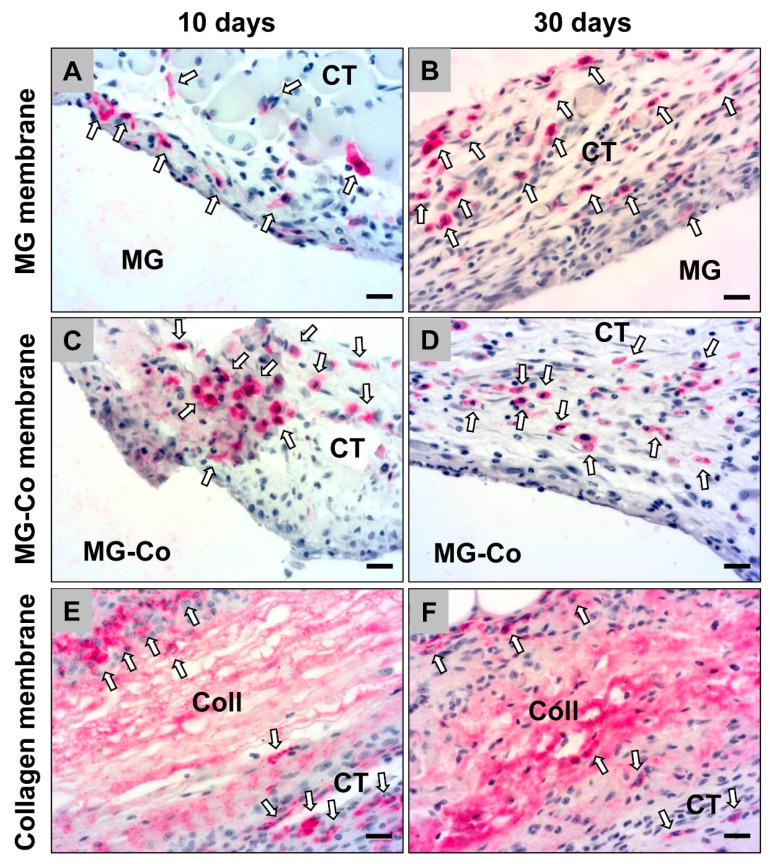
Exemplary histological images of anti-inflammatory (CD163-positive) macrophages (white arrows) within the implant beds of the different membranes, i.e., the pure magnesium membrane (MG) (**A**,**B**), the coated magnesium membrane (MG-Co) (**C**,**D**) and the collagen membrane (Coll) (**E**,**F**). CT = connective tissue (CD163 immunostainings, 400× magnification, scale bars = 10 µm).

The histopathological analysis of the occurrence of anti-inflammatory CD163-positive cells revealed that comparable numbers were detectable in the study groups of the pure MG membrane and the collagen membrane at day 10 post implantation (Figure 4). In the group of the coated MG membrane, lower numbers of this macrophage subtype were detectable at this early time point (Figure 4).

At day 30 post implantation, the histopathological analysis revealed that comparable numbers of CD163-positive macrophages were observable in all study groups (Figure 4). Thereby, tendentially lower numbers of anti-inflammatory macrophages were detected in the group of the collagen membrane (Figure 4).

**Figure 5:**
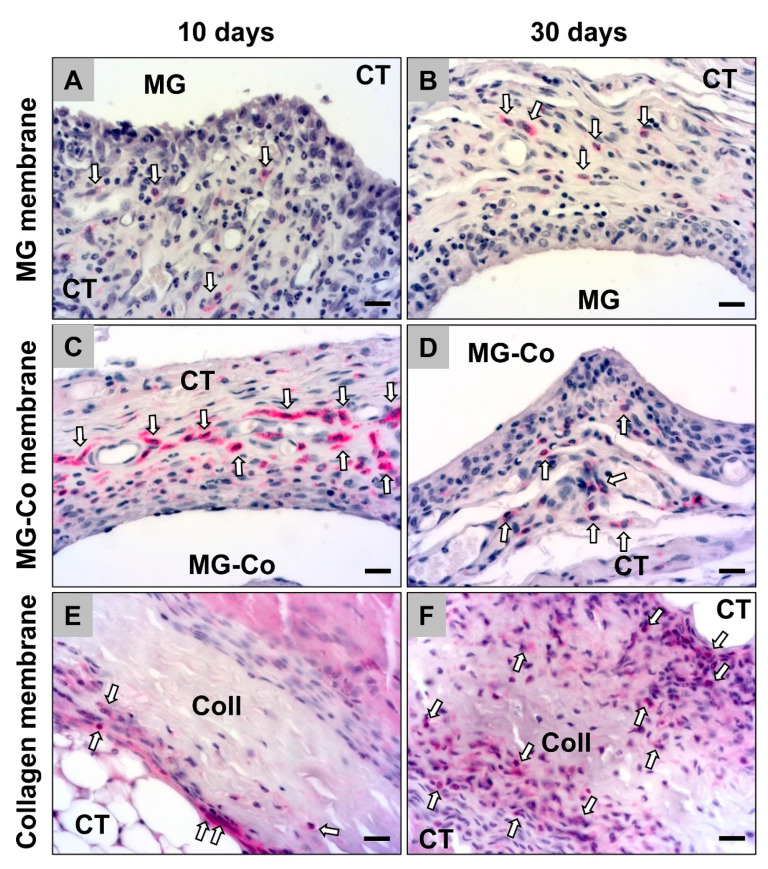
Exemplary histological images of pro-inflammatory (NF-κB-positive) macrophages (white arrows) within the implant beds of the different membranes, i.e., the pure magnesium membrane (MG) (**A**,**B**), the coated magnesium membrane (MG-Co) (**C**,**D**) and the collagen membrane (Coll) (**E**,**F**). CT = connective tissue (NF-κB immunostainings, 400× magnification, scale bars = 10 µm).

The histopathological analysis of the occurrence of pro-inflammatory NF-κB-positive cells showed that comparable numbers of this macrophage subtype were observable in the study groups of the pure MG membrane and the collagen membrane at day 10 post implantation, while lower numbers were detectable in the group of the coated MG membrane were detectable at this study time point (Figure 5).

The histopathological analysis revealed that comparable numbers of CD163-positive macrophages were still observable in all study groups at day 30 post implantation (Figure 5). Thereby, tendentially lower numbers of anti-inflammatory macrophages were still observed in the collagen membrane group (Figure 5).

### 3.3. Histomorphometric Analyses

**Figure 6:**
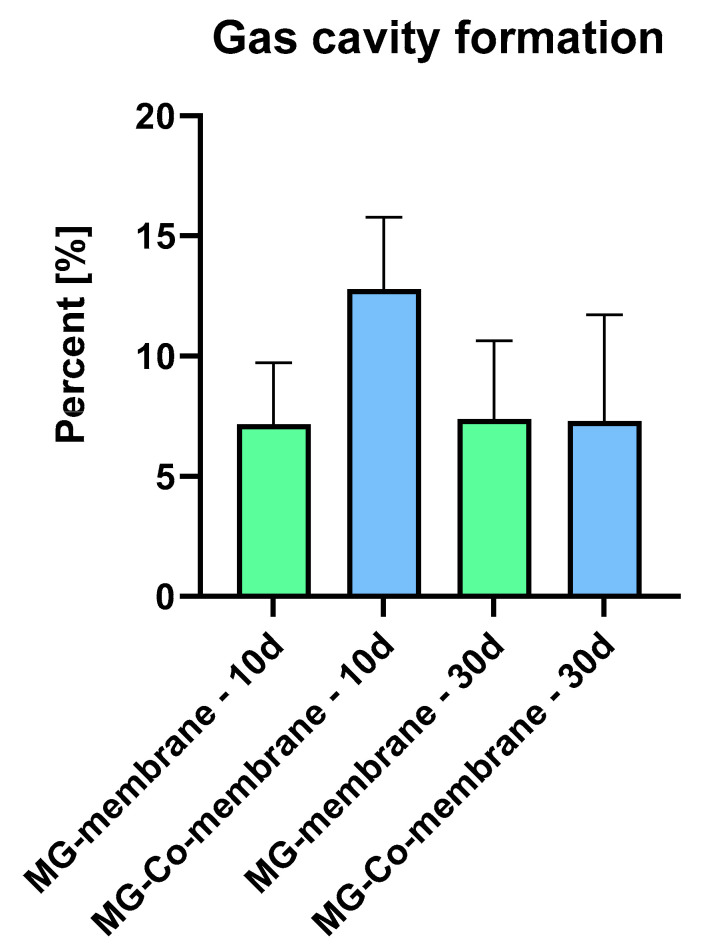
Histomorphometric results of the gas cavity formation in case of the analyzed magnesium-based membranes.

The histomorphometric analysis of the formation of gas cavities within the implant beds of the pure (MG) and the coated magnesium membranes (MG-Co) showed that comparable values were found at day 10 post implantation (MG: 7.16 ± 2.57%, MG-Co: 12.79 ± 2.98%), while the values in the MG-Co group were tendentially higher (Figure 4). At day 30 post implantation, also comparable values were found in both study groups (MG: 7.37 ± 3.27%, MG-Co: 7.29 ± 4.43%) (Figure 6).

**Figure 7:**
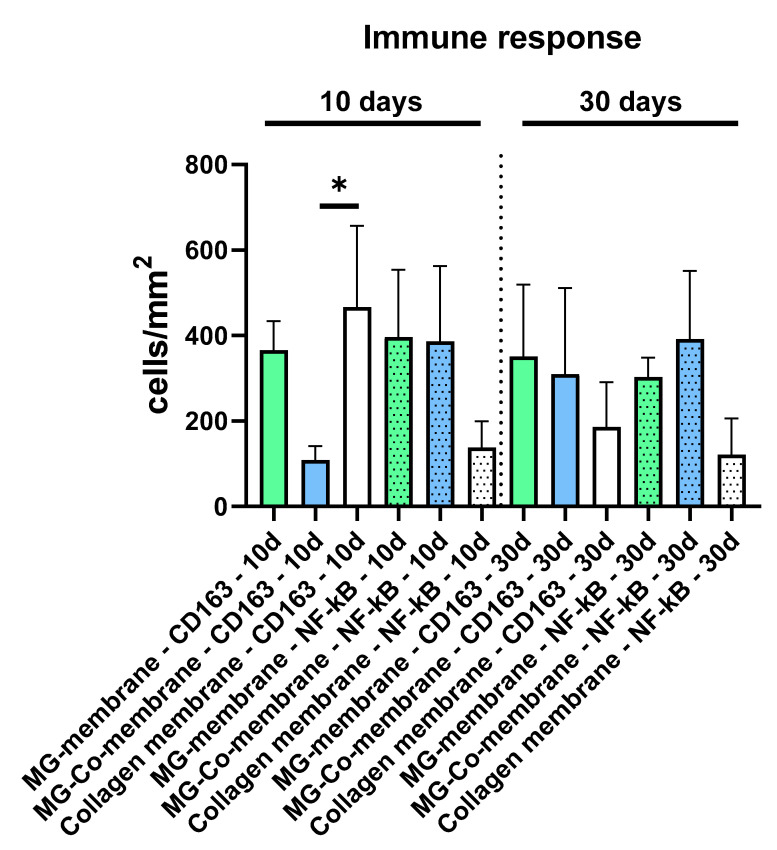
Histomorphometric results of the immune response, i.e., the detection of anti- (CD163 antigen) and pro-inflammatory macrophages (NF-κB antigen) at day 10 and day 30 post implantationem (separated by the dotted line) (* *p* < 0.05).

The histomorphometric analysis of the occurrence of the macrophage subtypes showed that comparable numbers of CD163-positive macrophages were found in the study groups of the pure MG membrane and the collagen membrane at day 10 post implantation, while lower numbers were found in the study group of the coated MG membrane at this early time point (Table 1 and Figure 7). Thereby, the numbers in the group of the coated MG membrane differed significantly compared to the numbers in the group of the collagen membrane (* *p* < 0.05), while no significant differences were found compared to the numbers in the group of the pure MG membrane (Table 1 and Figure 5). At this time point, the numbers of the NF-κB-positive cells did not significantly differ in all study groups (Table 1 and Figure 7). However, the lowest numbers were found in the group of the collagen membrane, while the numbers in the groups of both magnesium-based membranes were at a comparable level (Table 1 and Figure 7).

On day 30 post implantation, the histomorphometric analysis showed that the numbers of both the CD163-positive and the NF-κB-positive macrophages were comparable in all study groups, while tendentially lower numbers of pro- and anti-inflammatory macrophages were detected in the collagen membrane group (Table 1 and Figure 7).

## 4. Discussion

Bioresorbable membranes are the emerging alternative to non-resorbable membranes in the field of dentistry, especially in periodontal regenerative procedures, including guided bone or tissue regeneration (GBR/GTR) cases. Thereby, this membrane entity could also be used for other surgical specialties. Clinical relevance of biocompatible materials has gained importance from the clinicians as well as patients’ point of view in order to avoid additional surgical interventions as well as the reassurance of the usage of the human body’s degradable materials. Even collagen-based barrier membranes are nowadays mainly used for most of the oral standard defects representing successful and biocompatible medical devices [2,5,6,32,33]. However, they do not present the degree of volume stability that is required for multidimensional jaw defects [2,5,6,32,33]. In these cases still, nonresorbable membranes based on compounds like dense polytetrafluoroethylene (dPTFE) or expanded polytetrafluoroethylene (e-PTFE) in combination with titanium meshes as so-called titanium-reinforced PTFE membranes are the materials of choice, although their application requires a second surgical intervention with a variety of related side effects [4]. Thus, the development of resorbable volume-stable membranes is of special interest.

Among different other chemical compounds, Mg has been shown to constitute a favorable biomaterial for the development of stabilizing structures due to its satisfying biocompatibility, biodegradability and mechanical properties [5]. Furthermore, the combination of a magnesium mesh embedded in a native collagen membrane allows volume-stable interventions and complete biodegradation. Interestingly, previous studies confirmed the high level of biocompatibility with minimal inflammation, as well as the ability to support bone regeneration.

In this context, it has already been revealed that the combination of a magnesium mesh embedded in a native collagen membrane allows volume-stable interventions and complete biodegradation [5]. In this study, a hydrofluoric acid (HF)-treatment was analyzed that should prevent premature Mg degradation. The in vitro results of this study showed that the HF-treated Mg showed higher cytocompatibility, and HF-Mg prevented in vivo the formation of gas cavities. Thus, the HF-Mg meshes embedded in native collagen membranes were classified as a volume stable and biocompatible alternative to the non-absorbable synthetic materials.

In the present study, a new approach called ion implantation (II) with PVD coating was used for the passivation of Mg. In order to demonstrate comprehensive biocompatibility and successful passivation of the Mg membranes, untreated Mg (MG) and coated Mg (MG-Co) were investigated in vitro and in vivo. Thereby, in vitro tests were accomplished in accordance with EN ISO 10993-5/-12 regulations. In vivo, both membrane types were implanted using a manifoldly described subcutaneous implantation model in BALB/c mice for up to 30 days post implantation [2,5,6,21,22]. Furthermore, established histological, histopathological and histomorphometric analysis methods were applied that included the investigation of the M1-/M2-macrophage response to the biomaterial [5,21,22]. Both an uncoated Mg-based membrane and a collagen membrane (Jason membrane) with a previously described excellent biocompatibility were used as controls [5]. It must be noted that such studies, including the different steps of biomaterial testing in accordance with the development process following the respective guidelines, are of great importance prior to clinical trials. Especially studies analyzing material characteristics and preclinical studies allow examining the influence of (new) biomaterials on disease progression [34,35]. In this context, they also help to clarify the molecular basis of biomaterial applications [36,37,38].

In vitro, both membrane entities showed poor cytocompatibility with signs of massive gas bubble formation. In comparison, MG-Co showed inferior values than MG. Gas bubbles can be interpreted as a sign of degradation, which has been shown in previous publications [30,31]. However, the passivation seems to be an even triggering factor for degradation and the PVD coating was visible as black discoloration in the live–dead staining. The black discoloration can be attributed to the coating method. It is reasonable to assume that the layer is not stable in an aqueous environment and does not have a stabilizing effect in the form of slowed down degradation.

The results of the histopathological analysis showed that all membranes induced an inflammatory tissue reaction. The cells were mainly macrophages in addition to lower numbers of granulocytes and fibroblasts within the subcutaneous implantation beds. Interestingly, the MG-Co induced the highest extent of inflammation, including mainly macrophages directly attached to the material surfaces at this time point. The uncoated Mg-membrane induced a moderate level of inflammation, while the implantation of the collagen membrane was associated with the lowest level of inflammatory tissue response. In this context, it is known that collagen-based membranes show excellent biocompatibility without induction of major inflammatory responses [2,5,32]. Furthermore, it has already been revealed in different preclinical and clinical studies that the pericardium-based membrane used in the present study as a control device induces a tissue response that leads to the integration of the material involving even the cells of the collagen metabolism and also to a balanced M1-/M2-macrophage ratio needed for tissue regeneration in contrast to an inflammation-driven degradation [2,6,39].

Interestingly, these findings of poor cytocompatibility, the higher inflammatory response and the macrophage accumulation at the material surfaces indicate the involvement of the applied PVD coating in this process. It can be concluded that the induced macrophages contribute to the phagocytosis-driven degradation of the surface coating, as also found in the case of the aforementioned HF-treated Mg-membrane [5]. In this study, also an accumulation of macrophages in the group of the coated membranes was detected, and it was concluded that the HF-induced magnesium fluoride MgF2-layer was also phagocyted by macrophages in contrast to the pure Mg-meshes that induced only slight fibrosis, which has shown not to interfere with regenerative bone growth in a new study, but to serve as an osteoconductive scaffold (publication submitted).

Moreover, these findings are in line with further results in the present study. Thus, the histomorphometric data of the macrophage distribution revealed that the collagen membrane induced the highest numbers of CD163-positive M2-macrophages and the lowest numbers of NF-κB-positive M1-macrophages at day 10 post implantation. In contrast, the lowest numbers of M2-macrophages were found in the group of MG-Co, while the pure Mg-membrane induced a tissue response including a balanced number of M1- and M2-macrophages at this early time point. Furthermore, the analysis showed a trend towards a more pronounced M2-response in the groups of the collagen membrane and the pure MG-membrane at day 30 post implantation, while a trend towards a pro-inflammatory response was still visible in the MG-Co group. Altogether, these results reveal that the decreased the biocompatibility of the MG-Co membrane. However, the results furthermore show the good biocompatibility of the MG membrane as it induces similar results as also found in the collagen membrane group.

Finally, the histomorphometric analysis of the gas formation showed that the gas cavities occupied around 13% of the implant bed areas in the group of the MG-Co in contrast to around 7% in the group of the untreated Mg-membrane without significant differences at day 10 post implantation. However, no further differences were found at day 30 post implantation. Thus, in concordance with the in vitro results, the PVD coating seems to increase the degradation of the Mg-membrane instead of preventing the (initial) gas formation based on its absorbability of the H_2_ gas resulting from the membrane degradation. This observation may also be resulting from the observed higher immune response, including mainly pro-inflammatory macrophages, as this cell type is known for its phagocytic activity [6,22,23,33]. In this context, it is assumable that the higher macrophage accumulation, even in the case of MG-Co, led to increased degradation of the coating that had only a thickness of around 2 µm. Thus, the loss of the coating might have been led to the accelerated gas release.

Although the results of the present study show different tissue responses to the newly developed PVD coated Mg-membrane based on standardized research methods, it includes different limitations. First, the results of in vitro tests are limited as only standard methods based on ISO 10993-5/-12 were applied. In this context, the hydrogen release and the related (micro-) milieu conditions need to be applied, including special adaptions for the testing of Mg materials as already described by Jung et al. [29,30]. Furthermore, different other in vitro analysis approaches, such as innovative test setup, i.e., for example, CAD-CAM-based standardized sample models with equal shape and size, may provide deeper insights into cell-biomaterial interactions and improve the development of biomaterials already from this preclinical level [40].

Additionally, the implantation model, even in mice, is only used to show a general trend regarding the biocompatibility of a biomaterial. However, it cannot display the tissue reactions within the “target tissue”, i.e., the soft tissue of the oral cavity and the neighborhood to the underlying bone defect. Thus, it is assumable, especially the gas cavity formation differs after implantation within the oral cavity of larger animals like dogs or even in humans. This assumption is based on the fact that (a) hydrogen molecules can easily penetrate all tissue structures and quickly spread throughout the body due to their small atomic mass [30,31,41,42,43]. Furthermore, hydrogen gas is soluble in both water and fat at the same time [30,31,41,42,43]. These properties may ensure that the molecules can also penetrate fat layers or cell membranes and thus into fluid-filled cells. Additionally, the vascularization and hemodynamic, but also the tissue perfusion is higher, which should also lead to faster removal of the hydrogen gas. Furthermore, the counting of the M1- and M2-macrophages does not allow for the exact quantification of the immune response to a biomaterial and is thus only an initial indicator to assess the general material-related tissue response. For this purpose, specialized analysis methods, such as laser-assisted cell microdissection that enables the measurement of cytokine release from single cells, are more adequate tools for biomaterial research and development [44].

Taken together, the results of the present study revealed that the coated magnesium membranes did not show a significant benefit over the pure magnesium membrane. Furthermore, the additional PVD coating did not prevent gas formation but rather shows an acceleration. The pure magnesium membranes seem to represent a promising alternative compared to the actual generation of nonresorbable volume stable materials for GBR/GTR therapy. The present study showed that the pure magnesium membranes fulfill the requirements for biocompatibility even due to the comparability of the integration behavior of the collagen membrane.

## 5. Conclusions

Magnesium membranes represent a promising alternative compared to the nonresorbable volume of stable materials for GBR/GTR therapy. The present study showed that both the untreated and PVD-coated membranes were not cytocompatible, but the pure magnesium membranes fulfill the requirements for biocompatibility even due to the comparability of the integration behavior and the immune response of the collagen membrane. Interestingly, the PVD coating did not have a positive influence on the gas cavity formation or the improvement of the immune response compared to the uncoated membrane. Thus, the present study revealed that the pure magnesium membranes fulfill the requirements for biocompatibility and seem to represent a promising alternative compared to the actual generation of nonresorbable volume stable materials for GBR/GTR therapy.

## Figures and Tables

**Table 1:** Occurrence of pro- and anti-inflammatory macrophages in the three study groups (cells/mm^2^).

Time Point	MG Membrane		Coated MG Membrane		Collagen Membrane	
	CD163	NF-κB	CD163	NF-κB	CD163	NF-κB
Day 10	365.9 ± 67.75	396.6 ± 157.3	108.2 ± 32.84	386.6 ± 175.4	466.4 ± 190.3	137.9 ± 62.04
Day 30	351.3 ± 167.4	302.9 ± 45.54	310.1 ± 200.8	391.5 ± 159.4	186.5 ± 104.3	120.9 ± 85.64

## References

[1] Wang H.L., Boyapati L. (2006). “PASS” principles for predictable bone regeneration. Implant. Dent..

[2] Korzinskas T., Jung O., Smeets R., Stojanovic S., Najman S., Glenske K., Hahn M., Wenisch S., Schnettler R., Barbeck M. (2018). In Vivo Analysis of the Biocompatibility and Macrophage Response of a Non-Resorbable PTFE Membrane for Guided Bone Regeneration. Int. J. Mol. Sci..

[3] Trobos M., Juhlin A., Shah F.A., Hoffman M., Sahlin H., Dahlin C. (2018). In vitro evaluation of barrier function against oral bacteria of dense and expanded polytetrafluoroethylene (PTFE) membranes for guided bone regeneration. Clin. Implant Dent. Relat. Res..

[4] Fontana F., Maschera E., Rocchietta I., Simion M. (2011). Clinical classification of complications in guided bone regeneration procedures by means of a nonresorbable membrane. Int J. Periodontics Restor. Dent..

[5] Barbeck M., Kühnel L., Witte F., Pissarek J., Precht C., Xiong X., Krastev R., Wegner N., Walther F., Jung O. (2020). Degradation, Bone Regeneration and Tissue Response of an Innovative Volume Stable Magnesium-Supported GBR/GTR Barrier Membrane. Int. J. Mol. Sci..

[6] Gueldenpfennig T., Houshmand A., Najman S., Stojanovic S., Korzinskas T., Smeets R., Gosau M., Pissarek J., Emmert S., Jung O. (2020). The Condensation of Collagen Leads to an Extended Standing Time and a Decreased Pro-inflammatory Tissue Response to a Newly Developed Pericardium-based Barrier Membrane for Guided Bone Regeneration. In Vivo.

[7] Hornberger H., Virtanen S., Boccaccini A.R. (2012). Biomedical coatings on magnesium alloys—A review. Acta Biomater..

[8] Zhao D., Witte F., Lu F., Wang J., Li J., Qin L. (2017). Current status on clinical applications of magnesium-based orthopaedic implants: A review from clinical translational perspective. Biomaterials.

[9] Chen Z., Mao X., Tan L., Friis T., Wu C., Crawford R., Xiao Y. (2014). Osteoimmunomodulatory properties of magnesium scaffolds coated with beta-tricalcium phosphate. Biomaterials.

[10] Hiromoto S., Inoue M., Taguchi T., Yamane M., Ohtsu N. (2015). In vitro and in vivo biocompatibility and corrosion behaviour of a bioabsorbable magnesium alloy coated with octacalcium phosphate and hydroxyapatite. Acta Biomater..

[11] Boissonnet G., Chalk C., Nicholls J.R., Bonnet G., Pedraza F. (2020). Thermal Insulation of YSZ and Erbia-Doped Yttria-Stabilised Zirconia EB-PVD Thermal Barrier Coating Systems after CMAS Attack. Materials.

[12] Onder S., Calikoglu-Koyuncu A.C., Kazmanli K., Urgen M., Torun Kose G., Kok F.N. (2015). Behavior of mammalian cells on magnesium substituted bare and hydroxyapatite deposited (Ti,Mg)N coatings. New Biotechnol..

[13] Tian P., Liu X. (2015). Surface modification of biodegradable magnesium and its alloys for biomedical applications. Regen. Biomater..

[14] Yu K., Chen L., Zhao J., Li S., Dai Y., Huang Q., Yu Z. (2012). In vitro corrosion behavior and in vivo biodegradation of biomedical beta-Ca_3_(PO_4_)_2_/Mg-Zn composites. Acta Biomater..

[15] Ding Y., Wen C., Hodgson P., Li Y. (2014). Effects of alloying elements on the corrosion behavior and biocompatibility of biodegradable magnesium alloys: A review. J. Mater. Chem. B.

[16] Franz S., Rammelt S., Scharnweber D., Simon J.C. (2011). Immune responses to implants—A review of the implications for the design of immunomodulatory biomaterials. Biomaterials.

[17] Ballini A., Cantore S., Scacco S., Coletti D., Tatullo M. (2018). Mesenchymal Stem Cells as Promoters, Enhancers, and Playmakers of the Translational Regenerative Medicine 2018. Stem Cells Int..

[18] Ogle M.E., Segar C.E., Sridhar S., Botchwey E.A. (2016). Monocytes and macrophages in tissue repair: Implications for immunoregenerative biomaterial design. Exp. Biol. Med..

[19] Spagnuolo G., Codispoti B., Marrelli M., Rengo C., Rengo S., Tatullo M. (2018). Commitment of Oral-Derived Stem Cells in Dental and Maxillofacial Applications. Dent. J..

[20] Ballini A., Scacco S., Coletti D., Pluchino S., Tatullo M. (2017). Mesenchymal Stem Cells as Promoters, Enhancers, and Playmakers of the Translational Regenerative Medicine. Stem Cells Int..

[21] Flaig I., Radenković M., Najman S., Pröhl A., Jung O., Barbeck M. (2020). In Vivo Analysis of the Biocompatibility and Immune Response of Jellyfish Collagen Scaffolds and its Suitability for Bone Regeneration. Int. J. Mol. Sci..

[22] Sieger D., Korzinskas T., Jung O., Stojanovic S., Wenisch S., Smeets R., Gosau M., Schnettler R., Najman S., Barbeck M. (2019). The Addition of High Doses of Hyaluronic Acid to a Biphasic Bone Substitute Decreases the Proinflammatory Tissue Response. Int. J. Mol. Sci..

[23] Barbeck M., Booms P., Unger R., Hoffmann V., Sader R., Kirkpatrick C.J., Ghanaati S. (2017). Multinucleated giant cells in the implant bed of bone substitutes are foreign body giant cells-New insights into the material-mediated healing process. J. Biomed. Mater. Res. A.

[24] Barbeck M., Serra T., Booms P., Stojanovic S., Najman S., Engel E., Sader R., Kirkpatrick C.J., Navarro M., Ghanaati S. (2017). Analysis of the in vitro degradation and the in vivo tissue response to bi-layered 3D-printed scaffolds combining PLA and biphasic PLA/bioglass components—Guidance of the inflammatory response as basis for osteochondral regeneration. Bioact. Mater..

[25] Barbeck M., Najman S., Stojanovic S., Mitic Z., Zivkovic J.M., Choukroun J., Kovacevic P., Sader R., Kirkpatrick C.J., Ghanaati S. (2015). Addition of blood to a phycogenic bone substitute leads to increased in vivo vascularization. Biomed. Mater..

[26] Barbeck M., Unger R.E., Booms P., Dohle E., Sader R.A., Kirkpatrick C.J., Ghanaati S. (2016). Monocyte preseeding leads to an increased implant bed vascularization of biphasic calcium phosphate bone substitutes via vessel maturation. J. Biomed. Mater. Res. A.

[27] Kapogianni E., Barbeck M., Jung O., Arslan A., Kuhnel L., Xiong X., Krastev R., Friedrich R.E., Schnettler R., Fienitz T. (2019). Comparison of Material-mediated Bone Regeneration Capacities of Sintered and Non-sintered Xenogeneic Bone Substitutes via 2D and 3D Data. In Vivo.

[28] Barbeck M., Udeabor S.E., Lorenz J., Kubesch A., Choukroun J., Sader R.A., Kirkpatrick C.J., Ghanaati S. (2014). Induction of multinucleated giant cells in response to small sized bovine bone substitute (Bio-Oss) results in an enhanced early implantation bed vascularization. Ann. Maxillofac. Surg..

[29] Jung O., Smeets R., Porchetta D., Kopp A., Ptock C., Muller U., Heiland M., Schwade M., Behr B., Kroger N. (2015). Optimized in vitro procedure for assessing the cytocompatibility of magnesium-based biomaterials. Acta Biomater..

[30] Jung O., Smeets R., Hartjen P., Schnettler R., Feyerabend F., Klein M., Wegner N., Walther F., Stangier D., Henningsen A. (2019). Improved In Vitro Test Procedure for Full Assessment of the Cytocompatibility of Degradable Magnesium Based on ISO 10993-5/-12. Int. J. Mol. Sci..

[31] Jung O., Porchetta D., Schroeder M.L., Klein M., Wegner N., Walther F., Feyerabend F., Barbeck M., Kopp A. (2019). In Vivo Simulation of Magnesium Degradability Using a New Fluid Dynamic Bench Testing Approach. Int. J. Mol. Sci..

[32] Barbeck M., Lorenz J., Kubesch A., Bohm N., Booms P., Choukroun J., Sader R., Kirkpatrick C.J., Ghanaati S. (2015). Porcine Dermis-Derived Collagen Membranes Induce Implantation Bed Vascularization Via Multinucleated Giant Cells: A Physiological Reaction?. J. Oral Implantol..

[33] Ghanaati S. (2012). Non-cross-linked porcine-based collagen I-III membranes do not require high vascularization rates for their integration within the implantation bed: A paradigm shift. Acta Biomater..

[34] Marrelli M., Maletta C., Inchingolo F., Alfano M., Tatullo M. (2013). Three-point bending tests of zirconia core/veneer ceramics for dental restorations. Int. J. Dent..

[35] Zant E., Bosman M.J., Grijpma D.W. (2013). Materiomics—High-Throughput Screening of Biomaterial Properties.

[36] Moore L.B., Kyriakides T.R. (2015). Molecular Characterization of Macrophage-Biomaterial Interactions. Adv. Exp. Med. Biol..

[37] Kirkpatrick C.J., Wagner M., Köhler H., Bittinger F., Otto M., Klein C.L. (1997). The cell and molecular biological approach to biomaterial research: A perspective. J. Mater. Sci. Mater. Med..

[38] Hu W.J., Eaton J.W., Ugarova T.P., Tang L. (2001). Molecular basis of biomaterial-mediated foreign body reactions. Blood.

[39] Puisys A., Zukauskas S., Kubilius R., Barbeck M., Razukevičius D., Linkevičiene L., Linkevičius T. (2019). Clinical and Histologic Evaluations of Porcine-Derived Collagen Matrix Membrane Used for Vertical Soft Tissue Augmentation: A Case Series. Int. J. Periodontics Restor. Dent..

[40] Marrelli M., Pujia A., Palmieri F., Gatto R., Falisi G., Gargari M., Caruso S., Apicella D., Rastelli C., Nardi G.M. (2016). Innovative approach for the in vitro research on biomedical scaffolds designed and customized with CAD-CAM technology. Int. J. Immunopathol. Pharmacol..

[41] Frankel G.S., Samaniego A., Birbilis N. (2013). Evolution of hydrogen at dissolving magnesium surfaces. Corros. Sci..

[42] Gu X., Zheng Y., Cheng Y., Zhong S., Xi T. (2009). In vitro corrosion and biocompatibility of binary magnesium alloys. Biomaterials.

[43] Walker J., Shadanbaz S., Kirkland N.T., Stace E., Woodfield T., Staiger M.P., Dias G.J. (2012). Magnesium alloys: Predicting in vivo corrosion with in vitro immersion testing. J. Biomed. Mater. Res. B Appl. Biomater..

[44] Cheng L., Zhang S., MacLennan G.T., Williamson S.R., Davidson D.D., Wang M., Jones T.D., Lopez-Beltran A., Montironi R. (2013). Laser-assisted microdissection in translational research: Theory, technical considerations, and future applications. Appl. Immunohistochem. Mol. Morphol..

